# *Molinostrongylus longmenensis* n. sp. (Strongylida: Molineidae) in the bat *Scotophilus kuhlii* (Leach; Chiroptera: Vespertilionidae) from China

**DOI:** 10.3389/fvets.2024.1446743

**Published:** 2024-09-11

**Authors:** Hui-Dong Ju, Rui Jian, Shi-Yue Wang, Li-Yun Qin, Wei-Li Gao, Zhen Xu, Hong Zhang

**Affiliations:** ^1^College of Chemical Technology, Shijiazhuang University, Shijiazhuang, China; ^2^Hebei Key Laboratory of Intractable Pathogens, Shijiazhuang, China; ^3^College of Basic Medicine, Chengde Medical University, Chengde, China; ^4^College of Public Health, Zhengzhou University, Zhengzhou, China; ^5^Shijiazhuang Center for Disease Control and Prevention, Shijiazhuang, China; ^6^School of Medicine, Hebei University of Engineering, Handan, China

**Keywords:** bats, parasite, nematode, morphology, genetic data, phylogeny

## Abstract

A new species of nematode, *Molinostrongylus longmenensis* n.sp., parasite of the genus *Molinostrongylus*, is described based on specimens recovered from the small intestine of *Scotophilus kuhlii* Leach, 1822 (Chiroptera: Vespertilionidae) in Longmen County, Guangdong Province, China. To date, 135 species of bat-parasitic nematodes have been reported worldwide. Overall, 13 species belonging to seven genera in three families have been described in China. The new species is characterized by the presence of three ventral and three dorsal longitudinal cuticular ridges perpendicular to the body surface, which appear posterior to the cephalic vesicle and extend to the caudal bursa in males and the posterior end in females. The female tail has two medium-sized subventral conical processes of equal length, as well as one large dorsal conical process, and one thin spine, lateral alae that extend to the position of the vulva, with a fin-like ending. In addition, the new species was also characterized using molecular approaches, such as sequencing and analyzing the internal transcribed spacer 1 (ITS-1) of the ribosomal DNA.

## Introduction

The lesser yellow house bat, *Scotophilus kuhlii* Leach, 1822 (Chiroptera: Vespertilionidae), is a nocturnal insectivorous bat species that is mainly distributed in southern China and Southeast Asia. It inhabits buildings, dead fronds of palm plants, or the undersides of leaves. Nests under bridge piers can also be used as habitats. *S. kuhlii* is a dominant species in Guangdong Province, China (1–4). From January 2008 to April 2010, Professor Li Haiyan and her team (College of Life Sciences, Guangzhou University) conducted a comprehensive biodiversity survey of bats across Guangdong Province, China. This study was supported by the Natural Science Foundation of Guangdong Province (8151009101000005) (5). For our research, Professor Li's team provided the nematode specimens collected from the small intestine of *S. kuhlii*.

The specimens were identified as a new member of the genus *Molinostrongylus* (Skarbilovitch, 1934). Durette-Desset and Chabaud (6) classified the genus *Molinostrongylus* into five groups. The group *alatus* is characterized by two lateral alae, three small ventral ridges, and three small dorsal ridges, with the distal end of the spicules divided into two branches. The morphology of the specimen described enables us to categorize it within group *alatus*, but the new member has significant differences from other members of this group. Furthermore, the new species was characterized via molecular approaches by sequencing and analyzing the ITS-1 of the ribosomal DNA.

To date, 135 species of bat-parasitic nematodes have been reported worldwide, belonging to 4 orders, 13 families, and 26 genera. Only 13 species from seven genera in three families have been described in China, which are distributed in Henan, Yunnan, Guangdong, Guangxi, and Taiwan provinces (7, 8). Four species of parasitic nematodes have been reported in bats of the genus *Scotophilus*: *Molinostrongylus scotophili (*Ferenc, 1973) and *Capillaria vietnamensis* (Meszaros, 1973) parasites in *Scotophilus temminckii* (9) and *Rictularia rhinolophi* (Kagei and Sawada, 1977) (10) and *Seuratum indicum* (Dey, 2003) parasites in *Scotophilus heathi* (11).

Here, we report a new species of nematodes from *S. kuhlii*, thus contributing to the inventory of the helminth biodiversity of this host.

## Materials and methods

From January 2008 to April 2010, Professor Li Haiyan and her research team collected a variety of bat species. Their findings included two *Rhinolophus pearsonii* in Yingde City, 36 *Rhinolophus sinicus*, five *Scotophilus heathi* in Huizhou City, seven *Taphozous melanopogon*, five *Hipposideros armiger*, and 35 *S. kuhlii* in Longmen County. Those 35 individuals of *S. kuhlii* were captured using harp traps. Euthanasia of the collected bats was performed in accordance with the Laboratory Animal—Guidelines for Euthanasia (GB/T 39760-2021). Nematode specimens isolated from the intestine of this host were washed in physiological saline, fixed, and then stored in 75% ethanol. The specimens were provided to our laboratory for study.

For light microscopic studies, the nematodes were cleaned with lactophenol. Photomicrographs were captured using a Nikon^®^ digital camera coupled with a Nikon^®^ optical microscope. Drawings were performed using a Nikon microscope drawing attachment. For scanning electron microscopy (SEM), specimens were fixed in 4% formaldehyde solution, then post-fixed in 1% OsO4, dehydrated with ethanol and acetone, and critical point dried. The samples were coated with gold and examined using a Hitachi S-4800 scanning electron microscope at an accelerating voltage of 20 kV. Unless noted otherwise, measurements are given in micrometers (μm), with the range followed by the mean in parentheses. The nomenclature for the caudal bursa is based on Durette-Desset et al. (7) and Durette-Desset and Digiani (12). The synlophe was studied according to the method provided by Durette-Desset (13). The voucher specimens were deposited in the College of Life Sciences, Hebei Normal University, Hebei Province, China.

Two female specimens were randomly chosen for molecular analysis. According to the manufacturer's instructions, genomic DNA from each sample was extracted using a Column Genomic DNA Isolation Kit (Shanghai Sangon, China). The ITS-1 region was amplified by polymerase chain reaction (PCR) using the forward primer NC1 (forward: 5′-AACAACCCTGAACCAGACGT-3′) and the reverse primer NC5R (reverse: 5′-AATGATCCTTCCGCAGGTTCACCTAC-3′) (14). The cycling conditions were as described previously (15). The PCR products were examined on GoldView-stained 1.5% agarose gels and purified using the Column PCR Product Purification Kit (Shanghai Sangon, China). Sequencing was carried out using a Dye Deoxy Terminator Cycle Sequencing Kit (v.2, Applied Biosystems, California, USA) and an automated sequencer (ABI-PRISM 377). Sequences were aligned using ClustalW2. The DNA sequences obtained herein were compared (using the algorithm BLASTn) with those available in the National Center for Biotechnology Information (NCBI) database (https://www.ncbi.nlm.nih.gov) (16, 17).

Based on the ITS-1 sequence data, phylogenetic trees were constructed using maximum likelihood (ML) inference with MEGA 6.0 software. *Amidostomoides monodon* and *Amidostomoides acutum* (Strongylida, Trichostrongyloidea, and Amidostomatidae) were treated as the outgroup. The ingroup included 12 species of six genera in one family: Molineidae. We used a built-in function in the software MEGA 6.0 to select a best-fitting substitution model for the present sequences according to the Bayesian information criterion. The K2 (Kimura 2-parameter) + G model for the ITS-1 sequence data was identified as the optimal nucleotide substitution model. Reliabilities for the ML tree were tested using 1,000 bootstrap replications, and the BI tree was tested using 50 million generations, and bootstrap values exceeding 70% were shown in the phylogenetic tree (18, 19).

## Results

A total of 36 specimens of the genus *Molinostrongylus* (16 male and 20 female, sex ratios 4/5), were obtained from the small intestines of 15 host individuals among the 35 *Scotophilus kuhlii* examined. The overall prevalence of *Molinostrongylus* spp. in these bats was 43% (15 out of 35). No nematodes of the genus *Molinostrongylus* were detected in the other five species of bats (*R. pearsonii, R. sinicus, S. heathi, T. melanopogon*, and *H. armiger*) in this survey. A new species of nematode, *Molinostrongylus longmenensis* n.sp. is described based on 23 specimens (11 males and 12 females).

**Figure 1:**
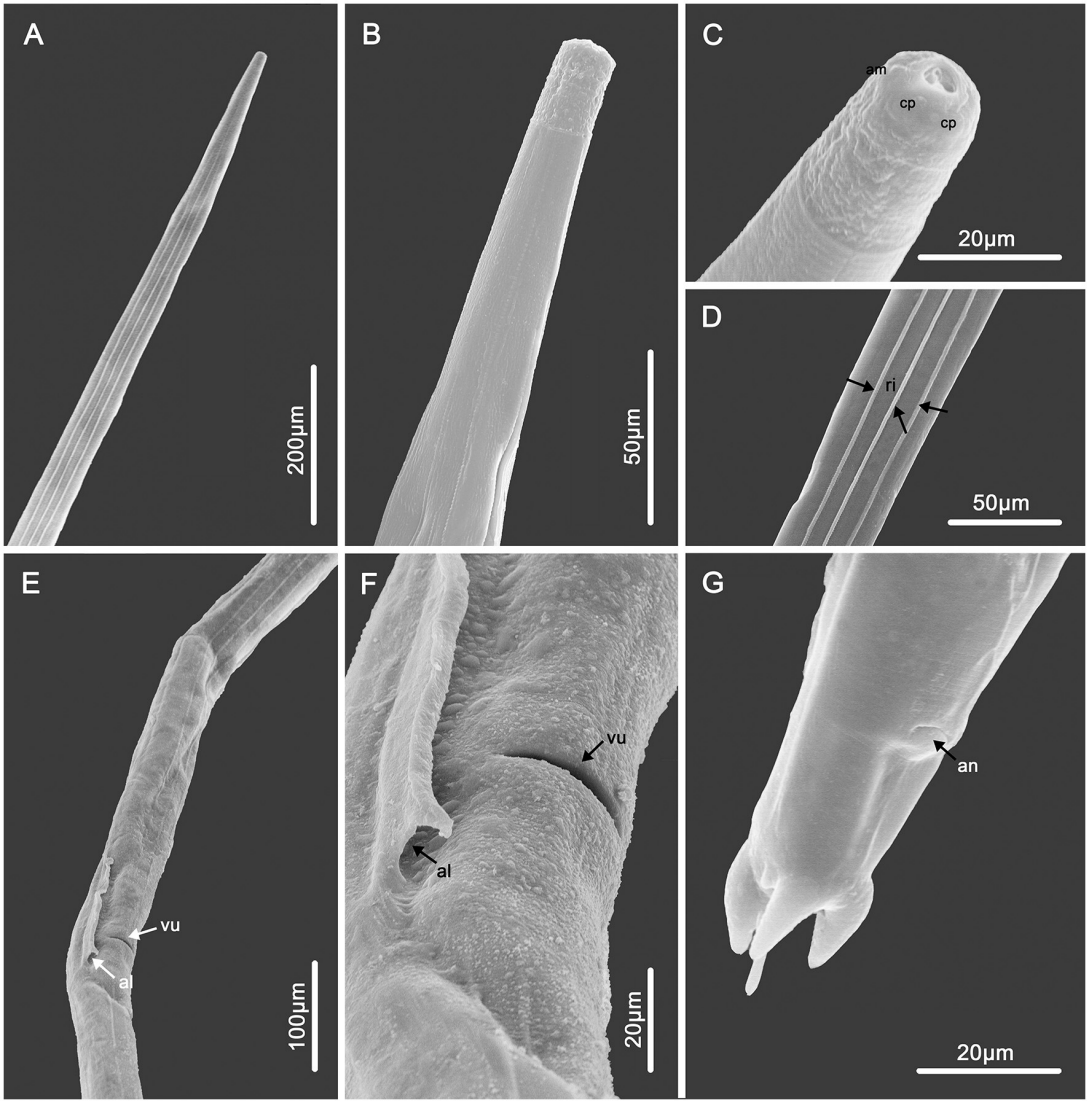
Scanning electron micrographs of female *Molinostrongylus longmenensis* n. sp. from *Scotophilus kuhlii*. **(A)** Anterior part, dorsal view. **(B)** Anterior part, ventral view. **(C)** Cephalic extremity, sub-apical view, amphid (am), and cephalic papillae (cp). **(D)** Mid-body, ventral view, showing the longitudinal ridges (arrows, ri). **(E)** Vulval region, lateral ventral view, vulva (arrow, vu), and the end of alae (arrow, al). **(F)** Magnified vulval region, vulva (arrow, vu), the end of alae (arrow, al). **(G)** Posterior end, lateral ventral view.

**Figure 2:**
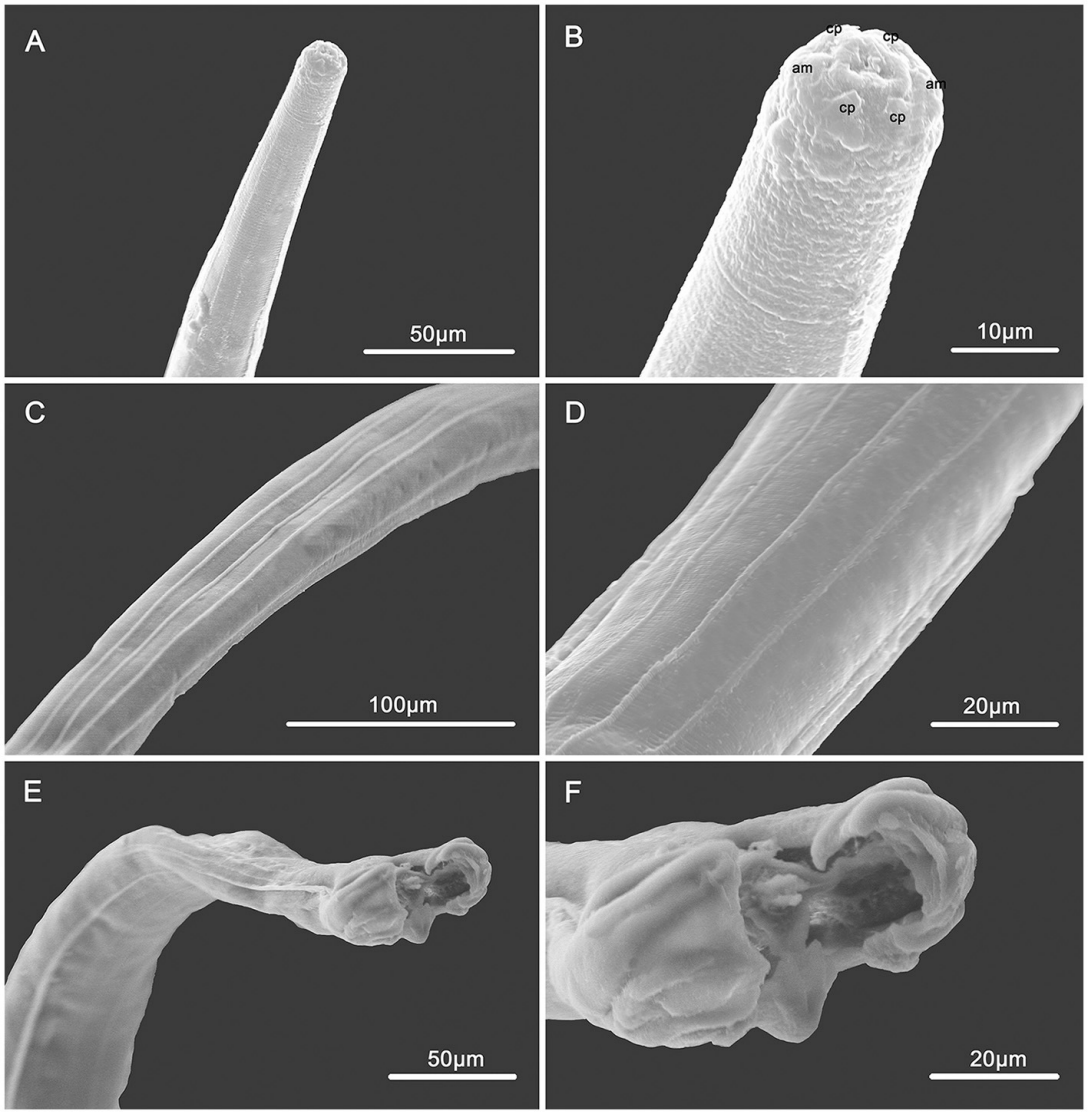
Scanning electron micrographs of male *Molinostrongylus longmenensis* n. sp. from *Scotophilus kuhlii* **(A)** Anterior part, lateral view. **(B)** Cephalic extremity, sub-apical view, amphid (am), and cephalic papillae (cp). **(C)** Mid-body, dorsal view, showing the longitudinal ridges. **(D)** Magnified mid-body, ventral view, showing the longitudinal ridges. **(E)** Posterior end, bottom view. **(F)** Caudal bursa, bottom view.

**Figure 3:**
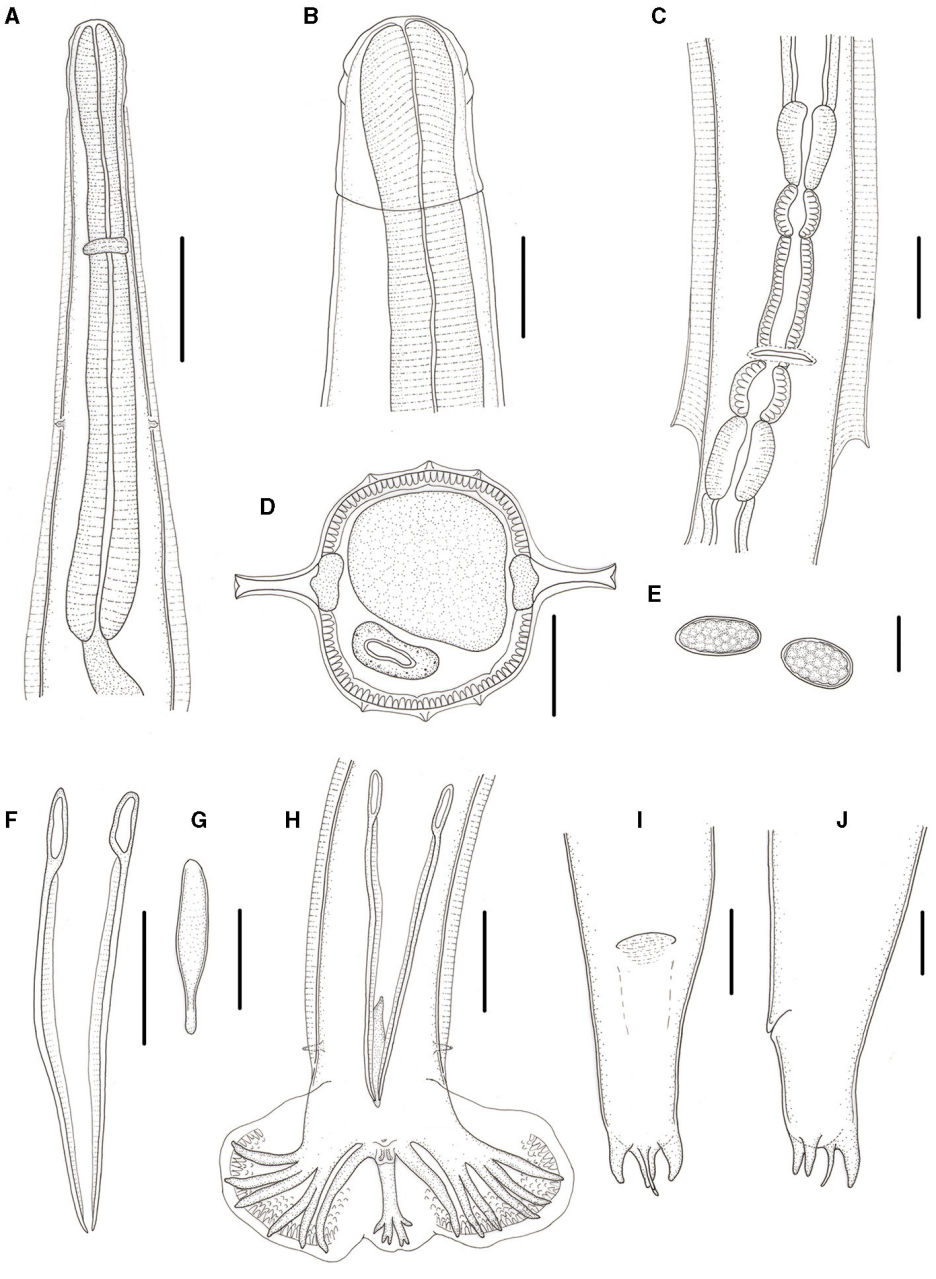
*Molinostrongylus longmenensis* n. sp. is collected from *Scotophilus kuhlii* (Leach; Chiroptera: Vespertilionidae) in China. **(A)** Anterior extremity, ventral view, male. **(B)** Anterior end, lateral view, female. **(C)** Region of vulva, ventral view. **(D)** Transverse section at mid-body, male. **(E)** Egg. **(F)** Spicules, ventral view. **(G)** Gubernaculums, ventral view. **(H)** Posterior end, ventral view, male. **(I)** Posterior end, ventral view, female. **(J)** Posterior end, lateral view, female. Scale bars **(A, C, E–J)** = 50 μm, **(B)** = 20 μm, and **(D)** = 30 μm.

**Figure 4:**
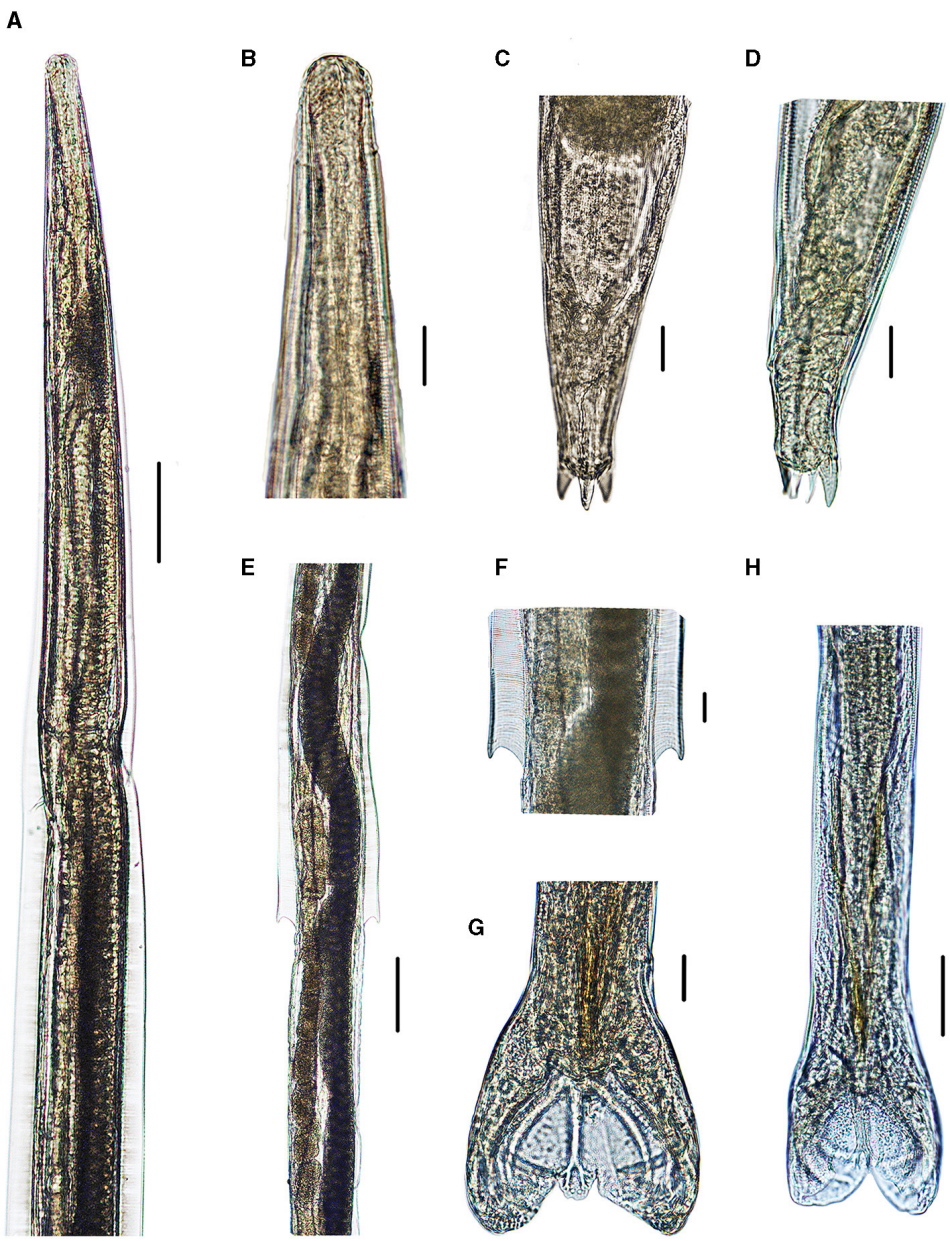
Photomicrographs of *Molinostrongylus longmenensis* n. sp. collected from *Scotophilus kuhlii* (Leach; Chiroptera: Vespertilionidae) in China. **(A)** Anterior extremity, ventral view, male. **(B)** Anterior end, lateral view, female. **(C)** Posterior end, dorsal view, female. **(D)** Posterior end, lateral view, female. **(E)** Vulval region, ventral view. **(F)** Magnified vulval region, ventral view. **(G)** Magnified posterior end of male, showing the dorsal ray. **(H)** Posterior end, ventral view, male. Scale bars **(A, E)** = 100 μm; **(B–D, F–H)** = 20 μm, and **(G)** = 50 μm.

*General*: Small-sized, whitish nematodes. Body cylindrical, maximum width at about region of middle. Cuticle with fine transverse striations. Cephalic vesicles distinct in both sexes (Figures 1B, C, 2B, 3B, 4A, B). Anterior extremity with a triradiate oral aperture, surrounded by a pair of amphids and four cephalic papillae (Figure 1C). Esophagus clavate, slightly broader posteriorly than anteriorly (Figure 3A). Lateral alae are present in both sexes. Lateral alae extending to caudal bursa in males (Figures 2E, 3H), and to the vulva, ending fin-like in females (Figures 1E, F, 3C, 4E, F). Nerve ring at approximately one-third of the esophageal length. Excretory pores situated at approximately two-third of esophageal length. Deirids slightly posterior to excretory pore (Figure 3A). Cuticular excretory furrow absent.

*Synlophe* (studied on one male and one female): In both sexes, the cuticle bears three ventral and three dorsal uninterrupted ridges, which appear posterior to the cephalic vesicle, extend to the caudal bursa in males (Figures 2A, C–F), and have a posterior end in females (Figures 1A, D, E). Longitudinal ridges are oriented perpendicularly to the body surface (Figure 3D). Three ventral and three dorsal ridges are nearly the same size in both males and females and are nearly parallel along the body.

*Male* (based on one holotype and nine paratypes): body 2.5–3.1 (2.8) mm long, maximum width 70–82 (75); cephalic vesicle 32–44 (35) long and 22–35 (28) wide; nerve ring, deirids, and excretory pore situated at 95–110 (103), 170–185 (177), and 165–176 (173), respectively, from anterior extremity; esophagus 240–285 (263) long, 30–50 (39) in maximum width; lateral alae extending to caudal bursa; three uninterrupted longitudinal cuticular ridges appear posterior to cephalic vesicle extending to caudal bursa. Caudal bursa symmetrical, lateral lobes with dotted ornamentation larger than dorsal lobe (Figures 2F, 3H, 4G, H). Pattern of caudal bursa being of type 3–2. Rays 3 and 4, rays 5 and 6 grouped with divergent extremities. Rays 2, 3, and 4 emerge together at the base of the trunk. Rays 3 and 4 bifurcated at the middle of the trunk. Rays 5 and 6 bifurcated at one-third of the trunk. Rays 2, 3, 4, 5, and 6 are almost equal in length with distal extremities almost reaching the bursal margin and directed ventrally. Ray 8 arises from the basis of dorsal ray, and is slightly longer than the latter. Dorsal ray bifurcates at the distal extremity into two branches, ray 9 arising first, and ray 10 divided into two branches (Figure 3H). Spicules relatively long and slender, length 169–181 (175) almost equal. Spicules with handles thicker and shorter than blade, blade with a striated ala extending from handle to tip of the blade (Figure 3F). Gubernaculum 39–50 (45) long, bowling pin shape, slightly curved in lateral view (Figure 3G). Genital cone well-developed with two presenting papillae 7 in each extremity, papillae zero presence (Figure 3H).

*Female* (based on 1 allotype and 10 paratypes): Body 3.0–4.2 (3.5) mm long, maximum width 80–95 (87). Cephalic vesicle 39–55 (45) long, 31–43 (36) wide. Nerve ring, deirids, and excretory pores situated at 132–147 (140), 200–221 (211), and 192–210 (202), respectively, from the anterior extremity. Esophagus 350–380 (358) mm in total length, 30–53 (40) in maximum width. Lateral alae 21–25 (23) in maximum width, extending to the vulva, and ending fin-like. Didelphic. Vulva transverse slit, 1.25–1.5 (1.35) mm from anterior extremity (Figures 1E, F). Vagina vera short and oriented perpendicular to the body surface, 15–22 (17) long, divided vestibule into two unequal parts, anterior part 50–92 (75) long, posterior part 10–22 (15) long. Anterior sphincter and infundibulum 30–45 (38) and 40–80 (62) long, respectively; posterior sphincter and infundibulum 39–46 (42) and 43–63(53) long, respectively (Figure 3C). Anterior uterine branch 0.8–1.2 (1.1) mm long, posterior uterine branch 0.8–1.3 (1.1) mm long. Each uterine branch with 8–15 eggs, the eggs are in the morula stage, irregularly oval, 50–98 (77) long and 29–65 (44) wide (Figure 3E). Anus 60–73 (68) from posterior extremity. Tail thick conical, with two medium-sized subventral conical processes equal in length, one large dorsal conical process, and one thin spine. Two of the conical processes equal in length, 13–18 (15) long; the other shorter one 6–9 (7) long. The thin spine 13–18 (15) in length (Figures 1G, 3I, J, 4C, D).

*Genetic characterization*: The three ITS-1 sequences of *M. longmenensis* n. sp., obtained in this study, are all 633 base pairs (bp) in length and correspond to a single genotype. Two ITS-1 sequences of *M. spasskii* obtained herein are all (632) bp in length and represent one genotype. A pairwise comparison of ITS-1 sequences between *M. longmenensis* n. sp. and *M. spasskii* displayed 1.58% nucleotide divergence. The ITS-1 sequences of *M. longmenensis* n. sp. and *M. spasskii* have been deposited in the GenBank database under the accession numbers PP853664–PP853666 (the sequences of *M. longmenensis* n. sp. are identical; the serial number is PP853664. The two ITS-1 sequences of *M. spasskii* are identical, depending on the collection location, respectively, PP853665 and PP853666).

**Figure 5:**
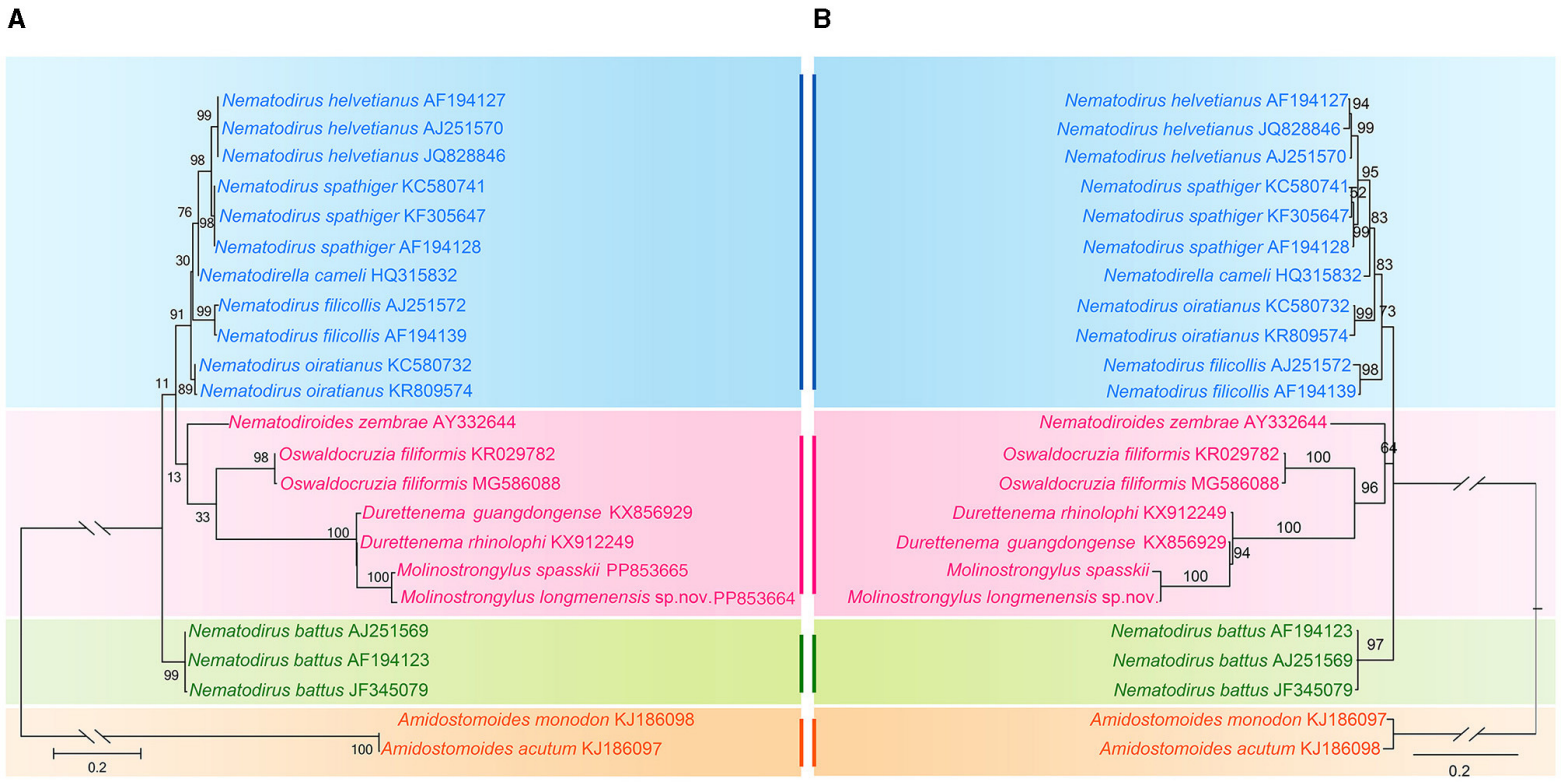
Phylogenetic relationships of representatives of the family Molineidae using maximum likelihood **(A)** and Bayesian inference **(B)** analyses based on the ITS-1 sequences. *Amidostomoides monodon* and *Amidostomoides acutum* (Strongylida, Trichostrongyloidea, and Amidostomatidae) was chosen as the outgroup. Bootstrap values exceeding 70% are shown in the phylogenetic trees.

Phylogenetic trees inferred from ML and Bayesian inference (BI) revealed that representatives of Molineidae were divided into three major clades (Figure 5). Clade I included the species of two genera, *Nematodirus* and *Nematodirella*; *Nematodirus* included four species, and *Nematodirella* included only one species (*Nematodirella cameli*). *Nematodirella cameli* displayed a closer relationship with *Nematodirus*. Clade II included the species of four genera, *Nematodiroides, Oswaldocruzia, Durettenema*, and *Molinostrongylus*. Among the four genera, *Durettenema* exhibited a closer phylogenetic relationship to *Molinostrongylus* than to *Nematodiroides* and *Oswaldocruzia*. Clade III included only *Nematodirus battus*.

### Taxonomic summary

*Molinostrongylus longmenensis* n. sp.

*Type host*: *Scotophilus kuhlii* Leach, 1822 (Chiroptera: Vespertilionidae).

*Site of infection*: Small intestine.

*Prevalence*: 43% (15 out of 35 dissected specimens were infected).

*Infection intensity*: 2–6 (2.4).

*Type locality*: Longmen County (23.65°N, 114.12°E), Guangdong Province, China.

*Type specimen deposition*: Holotype HBNU-M1007; Allotype HBNU-M1008; Paratypes (nine males, 10 females) HBNU-M1009. Deposited at the College of Life Sciences, Hebei Normal University, Hebei Province, China.

*Etymology*: The specific epithet refers to the type location, Longmen County.

## Discussion

The genus *Molinostrongylus* was established by Skarbilovitch in 1934 and *M. skrjabini* was described as the type species. To date, 20 species of the genus have been reported, including *M. ornatus* (Mönnig, 1927) (20), *M. pseudornatus* (Yeh, 1957) (21), *M. panousei* (Dollfus, 1954) (22), *M. dollfusi* (Thomas, 1958) (23), *M. heydoni* (Baylis, 1930) (24), *M. rhinolophi* (Yamaguti, 1941) (25), *M. richardae* (Durette-Desset and Chabaud, 1975), *M. bauchoti* (Durette-Desset and Chabaud, 1975), *M. benexae* Durette-Desset and Chabaud, 1975 (6), *M. alatus* (Ortlepp, 1932) (26), *M. skrjabini* (Skarbilovitch, 1934) (27, 28), *M. aelleni* (Durette-Desset and Chabaud, 1975) (6), *M. longispicula* (Yamashita and Mori, 1953) (29), *M. owyangi* (Durette-Desset and Chabaud, 1975) (6), *M. vespertilionis* (Morosov and Spassky, 1961) (27), *M. colleyi* (Durette-Desset and Chabaud, 1975) (6), *M. morosovi* (Andrejko, Pinchuk, and Skvorzov, 1968) (26), *M. scotophili* (Ferenc, 1973) (9), *M. tsuchiyai* Ohbayashi and Kamiya 1979 (30), and *M. spasskii* (Andrejko, Pinchuk, and Skvorzov, 1968) (23, 31).

Nematodes of the genus *Molinostrongylus* parasitic in bats, the general features include small sized; cephalic vesicle present; longitudinal ridges on ventral and dorsal sides extending from anterior to tail; wide lateral alae; amphids and cephalic papillae present; female tail with three conical processes and one thin spine; male caudal bursa consisting of two larger lateral lobes and one smaller dorsal lobe; rays 3 and 4 emerging together at the base of the trunk (6, 32). Characters of the present species are consistent with the features above.

Durette-Desset and Chabaud (6) classified the genus into five groups. Group *orantus*: two lateral alae, seven ventral ridges, and seven dorsal ridges; the distal end of spicules divided into two or three branches, parasitic in Miniopterina. Group *richardae*: two lateral alae, three large ventral ridges, and three large dorsal ridges, distal end of spicules divided into two branches, parasitic in Molossidae. Group *alatus*: two lateral alae, three small ventral ridges, and three small dorsal ridges, distal end of spicules divided into two branches, parasitic in the genus *Myotis* from Africa and Europe. Group *skrjabini*: two lateral alae, absence of dorsal and ventral ridges, distal end of spicules does not bifurcate or divide into two branches, parasitic in genre *Nyctalus*. Group *vespertilionis*: ***two*** wide and flat lateral alae, absence of dorsal and ventral ridges, distal end of spicules divided into two branches, parasitic in *Pipistrellus* in Europe and *Tylonycteris* in Malaisie (6, 33).

The present species is distinguished from the groups *orantus, richardae*, and *vespertilionis* in having three ventral ridges and three dorsal ridges instead of seven ventral ridges and seven dorsal ridges or no ridges. Moreover, group *vespertilionis* has *two* wide and flat lateral alae. Group *richardae* have the same pattern of ridges as our specimens, but they differ in the location of the excretory pore (anterior to the nerve ring *vs*. posterior to the nerve ring). Therefore, we assigned our specimens to group *alatus*. There are three species in group *alatus, M. alatus, M. spasskii*, and *M. scotophili* (Tables 1, 2). The present species differs from *M. alatus* by the significantly longer ray 9 and slightly longer ray 10. Moreover, the present species can be distinguished from *M. alatus* by having wide lateral alae extending to the vulva with a fin-like ending instead of extending to the posterior end.

**Table 1:** Morphometric data on male species of group *alatus* in the genus *Molinostrongylus*.

**Species**	***M. alatus***	***M. alatus***	***M. alatus***	***M. spasskii***	***M. scotophili***	***M. longmenensis* n.sp**.
Host	*Myotis chinensis*	*Myotis myotis*	*Myotis myotis*	*Pipistrellus abramus*	*Scotophilus temminckii*	*Scotophilus kuhlii*
Locality	Vietnam (9)	Bulgaria (26)	Suisse (6)	China (31)	Vietnam (9)	China
Length	3.3	3.54	2.8	3.1	2.7	2.8
Width	0.085	0.075	0.065	0.072	0.07	0.075
Cephalic vesicle	0.31	0.0385 × 0.031	0.035 × 0.03	0.035 × 0.032	0.035 × 0.028	0.035 × 0.028
Nerve ring	0.13	0.09	0.16	0.103	0.14	0.103
Deirids	0.14	0.0142	0.192	0.172	0.15	0.177
Excretory pore	0.16	0.0146	0.182	0.166	0.14	0.173
Esophagus	0.26	0.387	0.28	0.254	0.25	0.263
Type	–	–	3–2	3–2	–	3–2
Spicule	0.36	0.325	0.275	0.175	0.17	0.175
Gubernaculum	0.093	0.077	0.07	0.045	0.065	0.045

**Table 2:** Morphometric data on female species of group *alatus* in the genus *Molinostrongylus*.

**Species**	***M. alatus***	***M. alatus***	***M. alatus***	***M. spasskii***	***M. scotophili***	***M. longmenensis* n. sp**.
Host	*Myotis chinensis*	*Myotis myotis*	*Myotis myotis*	*Pipistrellus abramus*	Scotophilus temminckii	*Scotophilus kuhlii*
Locality	Vietnam (9)	Bulgaria (26)	Suisse (6)	China (31)	Vietnam (9)	China
Length	4–4.4	4.29	3	3.4	4.2–4.55	3.5
Width	0.1	0.093	0.1	0.089	0.1	0.087
Cephalic vesicle	–	0.0425 × 0.0376	0.035 × 0.03	0.047 × 0.036	0.037 × 0.031	0.045 × 0.036
Nerve ring	0.16	0.085	0.16	0.136	0.15	0.14
Deirids	0.17	189.5	0.19	0.176	0.16–0.17	0.211
Excretory pore	0.2	176.6	0.18	0.169	0.15–0.17	0.202
Esophagus	0.32	0.348	0.26	0.285	0.29	0.385
Vulva	–	1.18	0.725	1.35	0.7–1.2	1.35
Vagina	–	0.202	0.028	0.018	–	0.017
Vestibule ant.	–	–	0.078	0.065	–	0.075
Vestibule post.	–	–	0.01	0.036	–	0.015
Sphincter ant.	–	–	0.029	0.025	–	0.038
Sphincter post.	–	–	0.022	0.019	–	0.042
Infundibulum ant.	–	–	0.025	0.03	–	0.062
Infundibulum post.	–	–	0.021	0.029	–	0.053
Uterine ant.	–	–	–	1.1	–	1.1
Uterine post.	–	–	–	1.1	–	1,1
Egg	–	0.0793 × 0.0423	0.072 × 0.04	0.073 × 0.053	0.06–0.065 × 0.032–0.039	0.077 × 0.044
Anus	–	0.094	–	0.066	–	0.068
Process 1	–	0.014	–	0.017	–	0.068
Process 2	–	0.014	–	0.017	–	0.015
Process 3	–	–	–	0.017	–	0.007
Spine	–	0.022	–	0.019	–	0.015

The present species is distinguished from *M. spasskii* and *M. scotophili* in having two medium-sized subventral conical processes equal in length and one large dorsal conical process at the tip of the female tail instead of three almost equally stout conical processes (*M. spasskii*) or one dorsal, two subventral, and two small lateral conical processes (*M. scotophili*). *M. scotophili* is different from our specimens in the patterns of the caudal bursa being 2–3 instead of 3–2. Moreover, the synlophe of the present species is markedly distinguished from that of *M. scotophili* in having three ridges appearing posterior to the cephalic vesicle and extending to the caudal bursa in males and to the posterior end in females, and in having three longitudinal cuticular ridges appearing posterior to the cephalic vesicle and extending to the region of the vulva in the female, and extending to two-thirds of the body length in the male, then the number of these lines is doubled, that is, six in the last third of the body length.

Currently, the specific diagnosis of *Molinostrongylus* spp. is based mainly on morphology, and the genetic data of these parasites are severely limited. Based on the genetic analysis of *M. longmenensis* n. sp., no intraspecific nucleotide differences in the ITS-1 regions among different individuals were noted, but a high level of interspecific genetic variation in these regions among species of the other genera in the family Molineidae was clear.

Both morphological and genetic evidence supported the conclusion that our nematode specimens collected from *S. kuhlii* represent a new species of nematode parasite in bats. Bats are considered important reservoirs of pathogens of veterinary and medical relevance worldwide (34). Indeed, there is still an important lack of knowledge about parasites infecting bats. In this sense, further investigations into the parasitological fauna of bats are necessary, which could result in the description of numerous new species of helminths.

## Data Availability

The datasets presented in this study can be found in online repositories. The names of the repository/repositories and accession number(s) can be found at: https://www.ncbi.nlm.nih.gov/genbank/, PP853664–PP853666.
